
JOURNAL INFORMATION
==============================
NLM Title Abbreviation: Biospektrum (Heidelb)
Journal ID: Biospektrum (Heidelb)
NLM Journal ID: 9615685

Biospektrum

ISSN: 0947-0867
EISSN: 1868-6249

ARTICLE INFORMATION
==============================
PMCID: PMC8233606
PMID: 34219983
DOI: 10.1007/s12268-021-1587-3
Article ID: 1587
Article version: 1
Subjects: Wissenschaft

Atlas der SARS-CoV-2-RNA-Protein-Interaktionen in infizierten Zellen

Schmidt Nora 1
Munschauer Mathias mathias.munschauer@helmholtz-hiri.de http://www.helmholtz-hiri.de
1 2
1 grid.7490.a 0000 0001 2238 295X Helmholtz-Institut für RNA-basierte Infektionsforschung (HIRI), Helmholtz-Zentrum für Infektionsforschung (HZI), Josef-Schneider-Straße 2/D15, D-97080 Würzburg, Deutschland
2 grid.8379.5 0000 0001 1958 8658 Medizinische Fakultät der Julius-Maximilians-Universität Würzburg, Josef-Schneider-Straße 2/D15, D-97080 Würzburg, Deutschland
Electronic publication date: 2021 Jun 26
Print publication date: 2021
Volume: 27
Issue: 4
First page: 376
Last page: 379
Copyright: © Die Autoren 2021
License: Open Access: Dieser Artikel wird unter der Creative Commons Namensnennung 4.0 International Lizenz veröffentlicht, welche die Nutzung, Vervielfältigung, Bearbeitung, Verbreitung und Wiedergabe in jeglichem Medium und Format erlaubt, sofern Sie den/die ursprünglichen Autor(en) und die Quelle ordnungsgemäß nennen, einen Link zur Creative Commons Lizenz beifügen und angeben, ob Änderungen vorgenommen wurden. Die in diesem Artikel enthaltenen Bilder und sonstiges Drittmaterial unterliegen ebenfalls der genannten Creative Commons Lizenz, sofern sich aus der Abbildungslegende nichts anderes ergibt. Sofern das betreffende Material nicht unter der genannten Creative Commons Lizenz steht und die betreffende Handlung nicht nach gesetzlichen Vorschriften erlaubt ist, ist für die oben aufgeführten Weiterverwendungen des Materials die Einwilligung des jeweiligen Rechteinhabers einzuholen. Weitere Details zur Lizenz entnehmen Sie bitte der Lizenzinformation auf http://creativecommons.org/licenses/by/4.0/deed.de.
License URL: https://creativecommons.org/licenses/by/4.0/

issue-copyright-statement: © Springer-Verlag GmbH Deutschland, ein Teil von Springer Nature 2021

==============================
Using RNA antisense purification and mass spectrometry, we identified more than 100 human proteins that directly and specifically bind SARS-CoV-2 RNA in infected cells. To gain insights into the functions of selected RNA interactors, we applied genetic perturbation and pharmacological inhibition experiments, and mapped the contact sites on the viral RNA. This led to the identification of host dependency factors and defense strategies, which can guide the design of novel therapeutics against SARS-CoV-2.

■ Als obligat intrazelluläre Parasiten sind alle Viren von ihrer Wirtszelle abhängig, um sich zu vermehren. Das Virus zweckentfremdet die zelluläre Maschinerie, um seine Replikation voranzutreiben und schließlich neue Viruspartikel zu produzieren. Aus diesem Grund bieten Proteine der Wirtszelle, die zur Virusreplikation beitragen, gute Angriffspunkte für Medikamente. Die Wirtszelle wiederum verfügt über ein breites Repertoire an Abwehrmechanismen, um die Replikationsprodukte von eindringenden Viren zu erkennen und deren Vermehrung zu unterdrücken. Um wirksame Therapien für Viruskrankheiten entwickeln zu können, ist es daher essenziell, die Virus-Wirt-Interaktionen auf molekularer Ebene zu charakterisieren und zu verstehen.

Der Erreger der aktuellen COVID-19-Pandemie, SARS-CoV-2 (*severe acute respiratory syndrome coronavirus 2*), ist ein positiv-strängiges RNA-Virus, das sich im Cytoplasma der Wirtszelle vermehrt. Sämtliche genetische Information des Virus ist also in einem RNA-Strang enthalten. Dieses RNA-Molekül ähnelt zellulären Boten-RNAs (mRNAs) und trägt identische Modifikationen an seinen Enden. Nach Eintritt des Virus in die Zelle interagiert die virale RNA mit der zellulären Translationsmaschinerie, den Ribosomen, und nutzt diese für die Produktion viraler Proteine. Diese zuerst hergestellten viralen Proteine beinhalten Faktoren zur Transkription und Replikation des Virusgenoms. Über einen komplexen diskontinuierlichen Transkriptionsmechanismus generiert das Virus dann subgenomische mRNAs, welche als Vorlage für die Synthese von strukturellen Virusproteinen und somit zur Herstellung neuer Viruspartikel dienen.

Menschliche Zellen verfügen *per se* über hunderte RNA-Bindeproteine, die vielfältige Funktionen bei der Regulation von Transkription, Splicing, Transport, Translation, Stabilität und Abbau von RNAs ausführen (**Abb. 1**). In infizierten Zellen interagieren zelluläre RNA-Bindeproteine auch mit viraler RNA und können so zahlreiche Aspekte des Replikationszyklus von Viren maßgeblich regulieren (**Abb. 1**, [1]). Hieraus ergibt sich eine Abhängigkeit des Virus von bestimmten Wirtsfaktoren sowie die Funktion einer Vielzahl von RNA-Bindeproteinen als pro- oder antivirale Regulatoren. Viren verfügen beispielsweise nicht über eigene Ribosomen und sind daher vollständig auf die Translationsmaschinerie der Wirtszelle angewiesen, um virale Proteine zu synthetisieren.

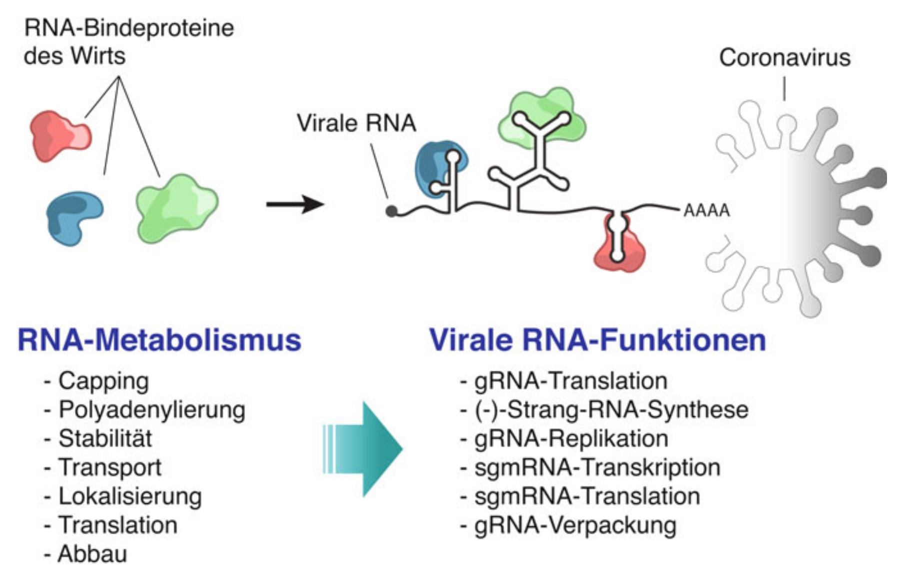

▲ **Abb. 1:** RNA-Bindeproteine des Wirts interagieren mit Virus-RNA und können so den Lebenszyklus und die Funktionen viraler RNA regulatorisch beeinflussen. gRNA: genomische RNA; sgm RNA: subgenomische mRNA.

## Identifizierung von RNA-Bindeproteinen mittels RAP-MS

Unsere Forschung konzentriert sich auf die RNA-Bindeproteine des Wirts, die direkte

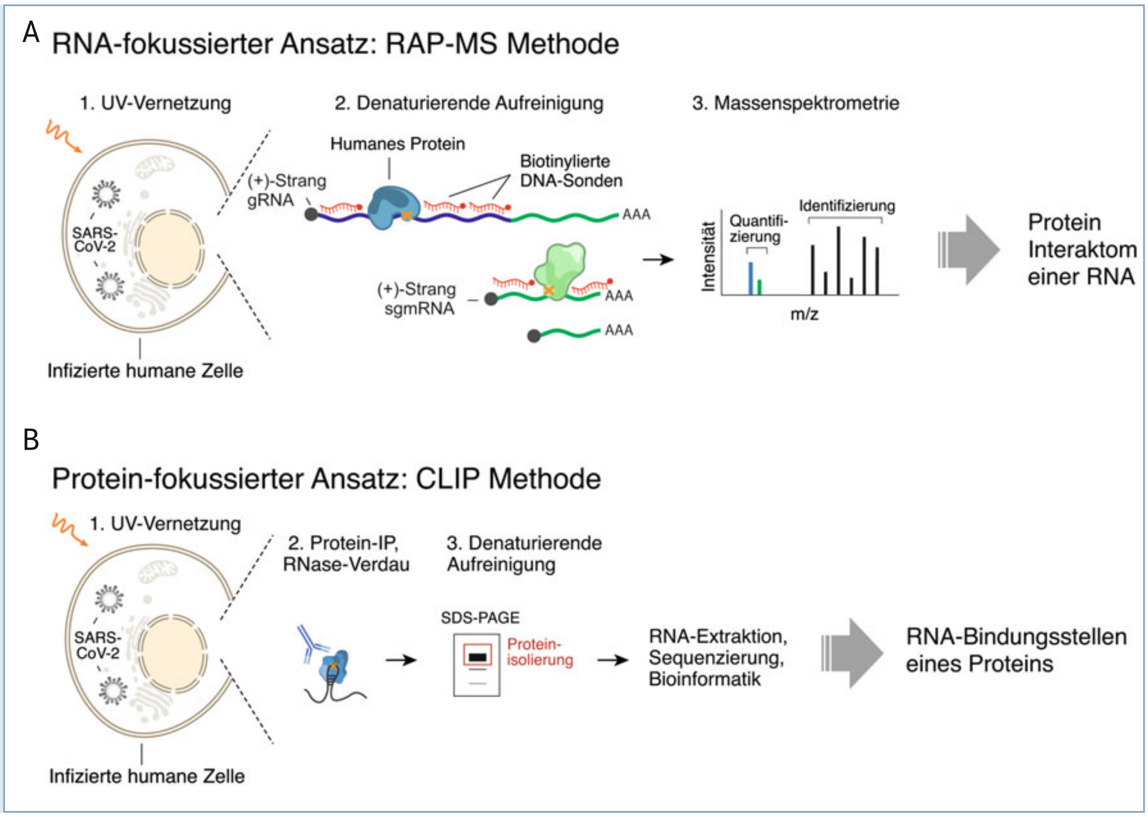

▶ **Abb. 2:** RNA- und Protein-fokussierte Ansätze zur Charakterisierung von RNA-Protein-Interaktionen in lebenden Zellen. **A**, Globale Identifizierung von direkten Interaktionspartnern einer Ziel-RNA mittels RAP-MS. **B**, Globale Kartierung von Protein-Bindungsstellen mittels CLIP-seq. IP: Immunpräzipitation.

und funktionelle Interaktionen mit der viralen RNA in mit SARS-CoV-2 infizierten Zellen eingehen. Um diese Proteine zu identifizieren, verwenden wir RAP-MS (*RNA antisense purification and mass spectrometry*) – eine Methode, bei der RNA selektiv angereichert wird, um die daran gebundenen Proteine durch Massenspektrometrie zu identifizieren (**Abb. 2A**, [2, 3]). Hierfür werden zunächst humane mit SARS-CoV-2 infizierte Zellen mit UV-Licht bestrahlt. Dadurch werden Proteine, die in direktem Kontakt mit RNA stehen, mit dieser kovalent vernetzt. Anschließend werden die Zellen unter denaturierenden Bedingungen lysiert und die SARS-CoV-2-RNA über sequenzspezifische DNA-Sonden aufgereinigt. Die Anwendung von denaturierenden Bedingungen während der Aufreinigung ist hierbei besonders wichtig, da so sichergestellt wird, dass nur direkt mit der RNA vernetzte Proteine erhalten bleiben, während indirekte Protein-Protein-Interaktionen aufgelöst werden. Die gebundenen Proteine werden schließlich mithilfe von quantitativen massenspektrometrischen Methoden identifiziert. Ein wichtiges Merkmal der RAP-MS-Methodik ist außerdem die Quantifizierung von RNA-Bindeproteinen relativ zu einer Kontroll-RNA mit bekannten Interaktionspartnern, wie beispielsweise der RMRP(*ribonuclease for mitochondrial RNA processing*)-Komplex.

## Virale und zelluläre Proteine interagieren direkt mit der SARS-CoV-2-RNA

In unserem experimentellen Ansatz haben wir humane Huh-7-Leberzellen mit SARS-CoV-2 infiziert. Mittels RAP-MS gelang es uns, über 100 Proteine zu identifizieren, die direkt und spezifisch an die virale RNA gebunden waren [4]. Darunter befanden sich erwartungsgemäß 15 virale Proteine, wie beispielsweise das Nucleocapsidprotein (N) und das Membranprotein (M), welche die virale RNA in neue Viruspartikel verpacken. Zusätzlich konnten wir zehn nicht strukturelle Proteine identifizieren, die u. a. für die Replikation und Transkription der viralen RNA benötigt werden. Die Wirtsproteine, die mit der SARS-CoV-2-RNA interagierten, enthielten wie erwartet eine große Anzahl an ribosomalen Proteinen und Translationsfaktoren. Eine nähere Betrachtung der biologischen Funktion der gefundenen Proteine zeigte, dass Regulatoren des RNA-Stoffwechsels in den Bereichen Transkription, Translation, oder RNA-Abbau in unserem Datensatz stark vertreten waren. Diese Ergebnisse belegten deutlich, dass unsere experimentelle Strategie aufging und wir in der Lage waren, direkte Interaktionspartner der SARS-CoV-2-RNA in infizierten Zellen zu identifizieren.

## Charakterisierung der RNA-Bindestelle durch CLIP-seq

Als komplementierenden Ansatz zu RAP-MS wurde die direkte Interaktion ausgewählter Proteine mit viraler RNA durch die CLIP-seq(*crosslinking and immunoprecipitation sequencing*)-Methodik validiert (**Abb. 2B**, [5]). Hierbei werden analog zu RAP-MS RNA und direkt daran gebundene Proteine in infizierten Zellen mit UV-Licht vernetzt. Anschließend wird hingegen nicht die virale RNA aufgereinigt, sondern ein ausgewähltes RNA-Bindeprotein durch einen spezifischen Antikörper präzipitiert. Die an das Protein gebundene RNA wird enzymatisch verdaut, wobei jener Teil, der direkt an das Protein gebunden ist, vom Verdau geschützt ist. Schließlich wird die proteingebundene RNA sequenziert. Diese Methode dient somit nicht nur der Validierung der durch RAP-MS identifizierten Bindeproteine, sondern gibt zusätzlich Aufschluss über die RNA-Sequenz im Interaktionsbereich mit dem Wirtsprotein. In unserem Fall konnten wir mittels CLIP-seq die Interaktionen von zwei RNA-

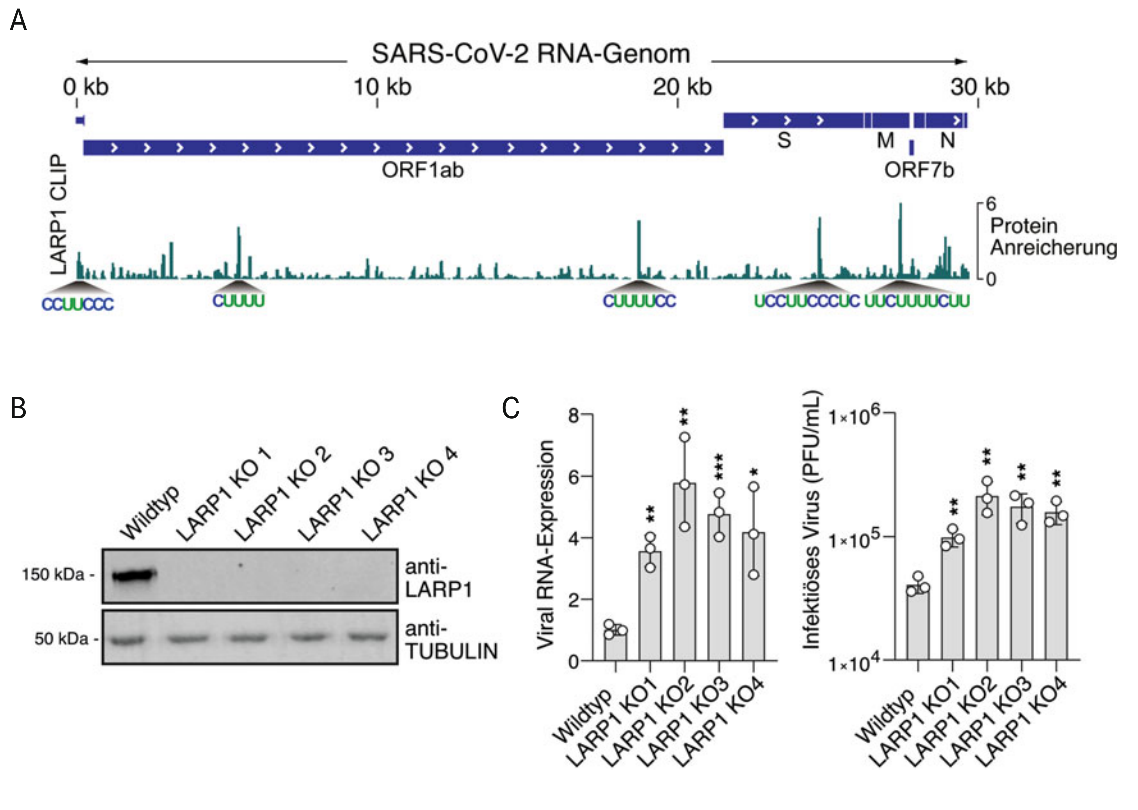

◀ **Abb. 3:** LARP1 bindet virale RNA und inhibiert die SARS-CoV-2-Replikation. **A**, Kartierung von LARP1-Bindungsstellen entlang des SARS-CoV-2-RNA-Genoms mittels CLIP-seq. **B**, Western Blot zeigt Inaktivierung des *LARP1*-Gens in vier unterschiedlichen Zellklonen. **C**, *LARP1*-Knockout(KO)-Zellen zeigen erhöhte Mengen an viraler RNA und infektiösem Virus nach SARS-CoV-2-Infektion. Abbildung aus [4].

Bindeproteinen, LARP1 und CNBP, mit der SARS-CoV-2-RNA bestätigen und die exakten Bindestellen hochauflösend kartieren [4].

## Depletion von SARS-CoV-2-RNA-Interaktoren beeinflusst die Virusreplikation

Ein Vergleich unserer Interaktionsdaten mit publizierten Ergebnissen aus globalen Knockout-Studien zeigte, dass mindestens elf der viralen RNA-Interaktoren einen pro- oder antiviralen Effekt in mit SARS-CoV-2 infizierten Zellen hatten [6]. Darunter befand sich interessanterweise auch CNBP, das am stärksten angereicherte Protein in unserem RNA-Interaktom-Datensatz. Frühere Arbeiten haben gezeigt, dass CNBP zur Freisetzung proinflammatorischer Zytokine bei Infektionen beiträgt und eine Rolle in der Translation strukturierter mRNAs haben kann [7, 8]. Um die Funktion von CNBP bei SARS-CoV-2-Infektionen weiter zu beleuchten, schalteten wir das Protein in Huh-7-Zellen genetisch aus und infizierten diese Zellen anschließend mit SARS-CoV-2. Hierbei detektierten wir deutlich höhere Mengen viraler RNA in CNBP-inaktivierten Zellen im Vergleich zu Kontrollzellen. Dies lässt darauf schließen, dass CNBP zur Unterdrückung der Virusreplikation in der Wirtszelle beiträgt.

## LARP1 bindet virale RNA an spezifischen Motiven

Ein weiteres stark angereichertes Protein in unserem RNA-Protein-Interaktom ist LARP1. LARP1 ist ein RNA-Bindeprotein, das die Translation bestimmter mRNAs reguliert und als Effektorprotein des mTOR-Signalwegs fungiert [9]. Es ist bereits bekannt, dass LARP1 zelluläre Transkripte mit TOP(*terminal oligopyrimidine*)-Motiven am 5′-Ende bindet und deren Translation inhibiert [9, 10]. Im Kontext der SARS-CoV-2-Infektion ergaben unsere CLIP-seq-Experimente, dass in viralen RNAs ebenfalls vergleichbare Oligopyrimidin-Motive von LARP1 gebunden werden (**Abb. 3A**, [4]). Interessanterweise fanden wir ein solches TOP-ähnliches LARP1-Bindemotiv auch in der 5′-*leader*-Sequenz, die alle SARS-CoV-2-mRNAs gemeinsam haben [11]. Zusätzlich zu der Interaktion zwischen LARP1 und viraler RNA haben wir auch einen regulatorischen Effekt von LARP1 auf die SARS-CoV-2-Replikation untersucht. In Zellen mit einem inaktivierten *LARP1*-Gen konnten wir ähnlich wie bei CNBP eine erhöhte Virusreplikation feststellen (**Abb. 3B**, **C**). Die Überexpression von LARP1 zeigte den entgegengesetzten Effekt und die Komplementierung von *LARP1*-Knockout-Zellen mit LARP1-Protein führte zu einer Reduktion der zunächst erhöhten Virusreplikation. Dies deutet auf einen antiviralen Mechanismus der Wirtszelle hin, wobei die Virusreplikation durch die direkte Bindung von LARP1 an die virale RNA unterdrückt wird. Wir vermuten, dass dies durch eine negative Regulation der Translation viraler Proteine durch LARP1 geschehen könnte. Weiterführende Arbeiten sind jedoch notwendig, um den zugrunde liegenden Mechanismus zu lösen.

## RNA-bindende Proteine als Angriffspunkte für antivirale Wirkstoffe

Für einige der von uns identifizierten SARS-CoV-2-RNA-Interaktoren existieren bereits zugelassene Wirkstoffe, die spezifisch die Aktivität dieser Proteine inhibieren. Um zu testen, ob unser Ansatz geeignet ist, um neue Angriffspunkte für antivirale Wirkstoffe zu finden, haben wir zunächst untersucht, ob die verfügbaren Inhibitoren Einfluss auf die Virusreplikation nehmen können. Zusätzlich zu dem beschriebenen Huh-7-Infektionsmodell wurde hierzu auch eine humane Lungenepithelzelllinie verwendet, also eine natürliche Zielzelle von SARS-CoV-2. Die Zugabe spezifischer Inhibitoren von drei unterschiedlichen RNA-Interaktoren (PPIA, ATP1A1 und ARP2/3) zum Zellkulturmedium während der Infektion konnte die Virusvermehrung signifikant reduzieren [4]. Dies zeigt nicht nur, dass die durch RAP-MS gefundenen Bindungspartner der viralen RNA relevant für die effiziente Vermehrung des Virus sind, sondern legt auch nahe, dass diese Wirtsproteine valide Ansatzpunkte für die Entwicklung antiviraler Therapien bieten könnten.

Der von uns gewählte Ansatz, Infektionen aus der Perspektive der viralen RNA und ihrer Interaktionspartner zu beleuchten, bietet also wichtige Einsichten hinsichtlich funktionell bedeutender Wirtsfaktoren und deren potenzieller Eignung als Zielstrukturen therapeutischer Wirkstoffe. Weiterführende Studien sind hier jedoch notwendig, um die Funktionen und molekularen Mechanismen der gefundenen RNA-Protein-Interaktionen genauer zu beleuchten. Dies wird essenziell zum Verständnis der SARS-CoV-2-Infektion auf molekularer Ebene beitragen und kann auf lange Sicht neue Wege zur Behandlung von COVID-19 aufzeigen. ■


Funding note: Open Access funding enabled and organized by Projekt DEAL.

Mathias Munschauer 2006–2010 Studium Biotechnologie, Hochschule Mannheim. 2010–2014 Promotion im Fach Biochemie, FU Berlin. 2014–2019 Postdoktorand am Broad Institute of MIT und Harvard. Seit 2019 Helmholtz-Nachwuchsgruppenleiter am Helmholtz-Institut für RNA-basierte Infektionsforschung. Seit 2021 Juniorprofessor an der Medizinischen Fakultät der Universität Würzburg.

Nora Schmidt 2009–2015 Studium der Technischen Biologie, Universität Stuttgart mit Masterarbeit am Imperial College London, UK, Labor von Prof. Dr. P. O’Hare. 2015–2019 Promotion im Bereich Virologie an der Universität Zürich, Schweiz, Arbeitsgruppe von Prof. Dr. B. Hale. Seit 2020 Postdoktorat in der Arbeitsgruppe von Jun. Prof. Dr. M. Munschauer am Helmholtz-Institut für RNA-basierte Infektionsforschung in Würzburg.


Literatur

[1] Garcia-Moreno M Jarvelin AI Castello A Unconventional RNA-binding proteins step into the virus-host battlefront Wiley Interdiscip Rev RNA 2018 9 e1498 10.1002/wrna.1498 30091184 PMC7169762
[2] McHugh CA Chen CK Chow A The Xist lncRNA interacts directly with SHARP to silence transcription through HDAC3 Nature 2015 521 232 236 10.1038/nature14443 25915022 PMC4516396
[3] Munschauer M Nguyen CT Sirokman K The NORAD lncRNA assembles a topoisomerase complex critical for genome stability Nature 2018 561 132 136 10.1038/s41586-018-0453-z 30150775
[4] Schmidt N Lareau CA Keshishian H The SARS-CoV-2 RNA-protein interactome in infected human cells Nat Microbiol 2021 6 339 353 10.1038/s41564-020-00846-z 33349665 PMC7906908
[5] Van Nostrand EL Pratt GA Shishkin AA Robust transcriptome-wide discovery of RNA-binding protein binding sites with enhanced CLIP (eCLIP) Nat Methods 2016 13 508 514 10.1038/nmeth.3810 27018577 PMC4887338
[6] Wei J Alfajaro MM DeWeirdt PC Genome-wide CRISPR screens reveal host factors critical for SARS-CoV-2 infection Cell 2021 184 76 91 10.1016/j.cell.2020.10.028 33147444 PMC7574718
[7] Chen Y Sharma S Assis PA CNBP controls IL-12 gene transcription and Th1 immunity J Exp Med 2018 215 3136 3150 10.1084/jem.20181031 30442645 PMC6279399
[8] Benhalevy D Gupta SK Danan CH The human CCHC-type zinc finger nucleic acid-binding protein binds G-rich elements in target mRNA coding sequences and promotes translation Cell Rep 2017 18 2979 2990 10.1016/j.celrep.2017.02.080 28329689 PMC5393907
[9] Philippe L van den Elzen AMG Watson MJ Global analysis of LARP1 translation targets reveals tunable and dynamic features of 5′ TOP motifs Proc Nat Acad Sci USA 2020 117 5319 5328 10.1073/pnas.1912864117 32094190 PMC7071917
[10] Fonseca BD Zakaria C Jia JJ La-related protein 1 (LARP1) represses terminal oligopyrimidine (TOP) mRNA translation downstream of mTOR complex 1 (mTORC1) J Biol Chem 2015 290 15996 16020 10.1074/jbc.M114.621730 25940091 PMC4481205
[11] Kim D Lee JY Yang JS The architecture of SARS-CoV-2 transcriptome Cell 2020 181 914 921 10.1016/j.cell.2020.04.011 32330414 PMC7179501
